# Comprehensive biomarker assessment for predicting severe acute kidney injury and need of kidney replacement therapy in liver transplantation patients

**DOI:** 10.1080/0886022X.2024.2402076

**Published:** 2024-09-17

**Authors:** Camila Lima, Gillene Santos Ferreira, Maria de Fátima Fernandes Vattimo, Luciana Bertocco de Paiva Haddad, Luiz Marcelo Malbouisson, Luiz Augusto Carneiro D’Albuquerque, Alexandre Toledo Maciel, Etienne Macedo

**Affiliations:** a Medical Surgical Nursing Department, School of Nursing, University of Sao Paulo, Sao Paulo, Sao Paulo, Brazil; b Department of Gastrointestinal Surgery, Clinical Surgery Division, University of Sao Paulo, Sao Paulo, Brazil; c Department of Anesthesiology, Clinical Surgery Division, University of Sao Paulo, Sao Paulo, Brazil; d Adult Intensive Care Unit, Research Department, Imed Group, Hospital Sao Camilo Pompeia, Sao Paulo, Brazil; e Nephrology Division, Department of Medicine, University of California San Diego, USA

**Keywords:** Acute kidney injury, liver transplantation, biomarkers, proenkephalin, neutrophil gelatinase-associated lipocalin

## Abstract

**Background:**

Renal dysfunction is a common complication following liver transplantation (LT). This study aimed to determine whether a comprehensive assessment of kidney function using nineteen serum and urinary biomarkers (BMs) within the first 48 h post-LT could enhance the prediction of severe acute kidney injury (AKI) and the need of kidney replacement therapy (KRT) during the first postoperative week.

**Methods:**

Blood and urine (U) samples were collected during the pre- and postoperative periods. Nineteen BMs were evaluated to assess kidney health in the first 48 h after LT. Classification and regression tree (CART) cross-validation identified key predictors to determine the best BM combination for predicting outcomes.

**Results:**

Among 100 LT patients, 36 developed severe AKI, and 34 required KRT within the first postoperative week. Preoperative assessment of U neutrophil gelatinase-associated lipocalin (NGAL) and liver-type fatty acid-binding protein (L-FABP) predicted the need for KRT with 75% accuracy. The combined assessment of U osmolality (OSM), U kidney injury molecule 1 (KIM-1), and tissue inhibitor of metalloproteinase (TIMP-1) within 48 h post-LT predicted severe AKI with 80% accuracy. U-OSM alone, measured within 48 h post-LT, had an accuracy of 83% for predicting KRT need, outperforming any BM combination.

**Conclusions:**

Combined BM analysis can accurately predict severe AKI and KRT needs in the perioperative period of LT. U-OSM alone proved to be an effective tool for monitoring the risk of severe AKI, available in most centers. Further studies are needed to assess its impact on AKI progression postoperatively.

Registered at Clinical Trials (clinicaltrials.gov) in March 24th, 2014 by title ‘Acute Kidney Injury Biomarkers: Diagnosis and Application in Pre-operative Period of Liver Transplantation (AKIB)’ and identifier NCT02095431.

## Introduction

Liver disease and cirrhosis are classified as the eleventh leading cause of death worldwide, representing a global challenge in the management of these pathologies [1]. In this context, liver transplantation (LT) is the final avenue of treatment for end-stage liver disease and is a great ally in reducing death rates [2,3]. Around 100,000 LTs are carried out annually worldwide, particularly in countries like the United States and Brazil [4].

Acute kidney injury (AKI) is a frequent postoperative complication of LT [5,6], associated with a significant impact on graft and patient survival [7,8]. The incidence of AKI in post-LT is higher than in other major noncardiac surgeries, and even patients with previously normal kidney function assessed by serum creatinine (sCr) are at great risk [9]. The most recent consensus on AKI diagnosis was published in 2012 by Kidney Disease: Improving Global Outcomes (KDIGO) [10] and is based on changes in sCr and urine output (UO). The International Club of Ascites (ICA) introduced new AKI diagnosis recommendations in 2012, updated in 2015 [11]. Unlike KDIGO, ICA criteria do not include the urine output criterion due to the frequent presence of oliguria in patients with preserved kidney function. Additionally, ICA subdivides KDIGO stage 1 into two subgroups based on sCr levels: stage 1 A (sCr <1.5 mg/dl) and stage 1B (sCr ≥1.5 mg/dl), with distinct outcomes supporting this subclassification.

Despite the improvement in the classification system, timely diagnosis of AKI in cirrhotic patients is challenging as sCr often underestimates kidney function in cirrhotic patients with decreased muscle mass, poor nutritional status, and volume overload [12]. Moreover, sCr is part of the mathematical model used to prioritize the allocation of LT: the Model End Stage Liver Disease (MELD) scoring system. Therefore, it is common for patients with higher MELD scores to present with impaired kidney function [13].

Several promising biomarkers (BMs) of early kidney dysfunction have been studied over the years, including in the LT setting [14–21]. Nevertheless, few studies evaluated a combination of BMs representing different dimensions of kidney function, such as filtration, reabsorption, secretion, concentration, kidney reserve, and fibrosis.

Given the many limitations of sCr in this scenario and considering that AKI is a complex and heterogeneous process, it is likely that a combination of BMs could improve AKI risk assessment, diagnosis, and prediction of severity. In this study, we aimed to determine whether a combination of BMs assessing kidney health could be a prognostic tool for determining the occurrence of severe AKI and need of kidney replacement therapy (KRT) in the first week after LT.

## Materials and methods

### Patients

During a 24-month period from 1 June 2013 through 31 June 2015, all planned LT recipients older than 18 years old were screened. Patients were enrolled into the study after voluntary informed consent was obtained per the Institution’s guidelines. The University of Sao Paulo Ethics Committee approved the study under protocol number: CAAE:06636513.4.0000.0068. The protocol was registered in Clinical Trials, available at https://clinicaltrials.gov by the identifier NCT 02095431 (24 March 2014). We excluded patients with need of KRT preoperatively, second LT, combined transplantation, CKD stage 5, or those with kidney transplantation [20,21].

### Data collection

We recorded baseline kidney function and comorbid history from electronic medical records (EMRs) from the admission for LT until 60 days after the transplantation. The following perioperative variables were included: main patient characteristics, intra-operative data, clinical follow-up in the first week after LT, need-for-KRT data, and outcomes.

The functional MELD was calculated based on serum bilirubin, international normalized ratio (INR), and sCr [22].

The standard triple-drug immunosuppression regimen of tacrolimus (calcineurin inhibitor) with mycophenolate mofetil and steroids was used to prevent allograft rejection.

Blood and urine samples were collected simultaneously preoperatively (before induction of anesthesia) and post-operatively (until 48 h after LT). This time frame was selected based on previous analyses to better assess the area under the curve (AUC) between 24 and 48 h [20,21]. Additionally, certain BMs in this study were measured across all these periods, with the maximum delta value within the 48-h window used for analysis. After collection, samples were immediately centrifuged: blood samples at 3000 rotations per minute (rpm) for 15 min, and urine at 1000 rpm for 10 min and stored at −80 °C until analysis.

### Clinical outcomes

The primary outcome was AKI development during the first week of LT based on the KDIGO sCr criteria [23]. Baseline kidney function was defined as the lowest value of sCr in the 3 months before LT and was used to assess kidney recovery. We considered reference sCr as the lowest value in the week before LT and this value was used to determine AKI diagnosis and staging. AKI stage was defined according to KDIGO: stage 1 (1.5 − 1.9 times sCr reference, or increase = >0.3 mg/dl until 48 h), stage 2 (2.0–2.9 times sCr reference), stage 3 (3.0 times sCr reference or an increase to >4.0 mg/dl) [14]. Patients with KDIGO 3 were categorized as severe AKI group.

### Biomarker’s assessment

The summary of the BMs evaluated is available in Table 1. Urine samples were centrifuged at 1000 rotations per minute (rpm) for 10 min to settle debris. The supernatant was diluted 400-fold and assayed for biomarker Alpha 1-Microglobulin (a-1M) using ABCAM kit (Ab226899). Urine samples were diluted 200-fold and albumin (ALB) was measured using Abnova (KA0455) kit (Walnut, CA, USA). Urine kidney-injury-molecule (U-KIM-1) and urine interleukine-18 (U-IL-18) were assayed using the luminex analyze by Milliplex^®^ (Darmstadt, Germany). Biomarker Cystatin C was measured in urine (U-CYS) (diluted 5-fold) and plasma (P-CYS) (diluted 50-fold) using BioLegend (445507) kit (San Diego, CA, USA). QuantiChrom^TM^ Urea Assay Kit (DIUR-100) from Bioassay Systems (Hayward, CA, USA) was used to measure urea in 5 ul of sample (urine/plasma). Blood urea nitrogen (BUN) was measured in (mg/dl) using the following formula: [Urea]/2.14. (1 mg/dl urea equals 167 μM, 0.001% or 10 ppm). The Sphingotest^®^ penKid^®^ immunoassay kit PEK96 (San Diego, CA, USA) measures Proenkephalin 119–159 in plasma, a stable surrogate for the kidney stimulating hormone enkephalin by immunoluminometric assay in pmol/l. Monocyte chemotactic protein-1 (U-MCP-1) was assayed after diluting urine samples 1:1 using R&D systems^®^ biotechne CCL2/MCP1 (DCP100) immunoassay (Minneapolis, MB, USA). U-CYS, U-ALB and U-a-1M were adjusted to urine creatinine. The concentrations of uromodulin in urine samples (U-UMOD) was measured using a Sigma-Aldrich ELISA kit (catalog number RAB0751), according to the manufacturer’s protocol. The urine and plasma levels of neutrophil gelatinase-associated lipocalin (NGAL) were measured with the Particle-enhanced turbidimetric immunoassay (PETIA)© and tests were performed in the Labmax 560 equipment. Urinary glutathione S-transferases (GST-pi), tissue inhibitor of metalloproteinase 1 (TIMP-1) and liver-type fatty acid-binding protein (L-FABP) were assayed using the luminex analyze by Milliplex^®^. The urine osmolality (U-OSM) levels were measured using the cryoscopic lowering method in Advanced Instruments (model 3320). All measures were blinded to the investigators.

**Table 1.** Biomarkers and their functional status and pathophysiology.

Marker type	Biomarker	Functional	Pathophysiology
Filtration	P-CYS	Filtration	Constantly produced by all nucleated cells, freely filtered by glomerulus, reabsorbed, and metabolized in the proximal tubular cells.
Filtration	P-PENK	Filtration	Established as a reliable surrogate marker for enkephalin. Due to its low molecular weight, it is freely filterable through the glomerulus.
Injury	U-KIM-1	Tubular Injury	Transmembrane glycoprotein produced after ischemic /nephrotoxic insults. It is increased in AKI and associated with CKD progression (fibrosis).
Injury	U P-NGAL	Tubular Injury	Secreted polypeptide that is upregulated in response to tubular injury and rapidly detectable in plasma and urine.
Injury	U-IL-18	Tubular Inflammation	Pro-inflammatory cytokine: increased in ischemic and inflammatory AKI
Injury	U-CYS	Tubular Injury	The presence of CYS in urine reflects tubular dysfunction, ischemic injury, GFR decline and CKD progression.
Stress	U-GST-pi	Tubular stress	Cytosolic enzyme soluble in cytochrome, produced in the distal tubule, which plays a role in detoxifying free radicals.
Stress	U-L-FABP	Tubular stress	Protein that participates in the transport of fatty acids to mitochondria and peroxisomes, which will be metabolized to reduce oxidative stress.
Reabsorption	U- a-1M	Reabsorption	Low molecular weight protein, filtered at the glomerulus but almost fully reabsorbed by proximal tubular epithelial cells, where it is degraded. Its presence reflects proximal tubular damage.
Reabsorption	U-ALB	Permeability	Filtered by glomerulus, reabsorbed and metabolized in the proximal renal tubule. Predictor of cardiovascular risk and CKD progression.
Secretion	FeNa	Secretion	Based on premise that intact tubules could reabsorb Na and injury tubules do not.
Secretion	FeU	Secretion	Based on premise that intact tubules could reabsorb Urea and injury tubules do not.
Secretion	FePCS	Secretion	Metabolite produced by intestinal bacteria that is eliminated *via* renal secretion. Its decrease also reflects tubular dysfunction and is associated with cardiovascular disease and CKD progression.
Secretion	FeHA	Secretion	Handled by organic anion transporters on the basolateral membrane. Decreased secretion reflects tubular dysfunction. Low excretion has been associated with CKD progression.
Concentration	U-OSM	Concentration	It measures the amount of solutes versus solvents in the urine.
Repair-Fibrosis	U-MCP-1	Fibrosis	Member of the chemokine family – regulates trafficking of monocytes in response to inflammatory signals. It is increased in ischemia – reperfusion injury.
Fibrosis	U-TIMP-1	Fibrosis	Physiological inhibitor of enzymes that degrade the collagen matrix, considered a BM of fibrosis.
Repair	U-UMOD	Tubular Reserve	Protein exclusively produced in the kidney by cells of the thick ascending limb of the loop of Henle and distal convoluted tubule.High levels of protein are associated with reduced mortality, CKD and AKI.

P: plasma; U: urinary; CYS: cystatin C; GFR: glomerular filtration rate; AKI: acute kidney injury; CKD: chronic kidney disease; BM: biomarker; PENK: pro-enkephalin; IL-18: interleukine-18; KIM-1: kidney-injury-molecule; NGAL: neutrophil gelatinase associated a lipocalin; a-1M: alpha 1 microglobulin; L-FABP: liver-type fatty acid-binding protein; GST-pi: glutathione S-transferases; ALB: albumin; (Fe): fraction excretion; Na: sodium; U: urea; PCS: p-creasol sulfate; HA: hippuric acid; OSM: osmolality; MCP-1: monocyte chemotactic protein-1; TIMP-1: tissue inhibitor of metalloproteinase; UMOD: uromudolin.

To calculate the fractional excretion of urea (FeU), sodium (FeNa), hippuric acid (FeHA), and p-creasol sulfate (FePCS), the following formula was used: (Urine BM x sCr)/(serum BM x urine creatinine) x 100.

### Statistical analysis

Continuous variables were presented as mean ± SD or median (25th − 75th percentiles) and were compared using one-way ANOVA or the Kruskal-Wallis test according to the Gaussian distribution. Kolmogorov-Smirnov test was used to check normality. Categorical variables are presented as absolute numbers and percentages and were compared by the Chi-square test. P values were two-tailed, and p = <0.05 was considered significant. The model fit using standard statistical measures such as the Hosmer-Lemeshow test, calibration plots, and discrimination measures such as the area under the receiver operating characteristic curve (AUC-ROC). The SPSS (*Statistical Package for Social Sciences*) version 20 (Chicago, Illinois, USA) software was used for the statistical analysis.

The classification and regression tree (CART) was used to identify the best BMs to assess the risk for AKI and the need for KRT. The CART analysis performed a joint regression with the 19 analyzed BMs. The first BM (the ‘mother node’), is the best to stratify the group, providing the cutoff value and sensitivity (S), specificity (E), and accuracy. Later nodes are called ‘child nodes’. The objective is to achieve the best performance of the classification resulting in a decision tree that can be used as a visual and analytical decision support tool. One of the main advantages of using decision trees is that the process will automatically include in its rule only the BMs that matter in deciding and BMs which are not important or relevant will be ignored. The predictive variables for outcomes that were selected in the CART analysis were: plasma (P) and urine (U)-CYS, P-PENK, U-IL-18, U-KIM-1, P-NGAL, U-NGAL, U-CYS, U-a-1M, FeNa, FeU, FePCS, FeHA, U-MCP-1, U-TIMP, U-L-FABP, U-OSM, and U-UMOD. CART analysis was performed using the R ‘tree’ package in R (version 3.3.1, 2016).

## Results

### Cohort characteristics

**Figure 1.**
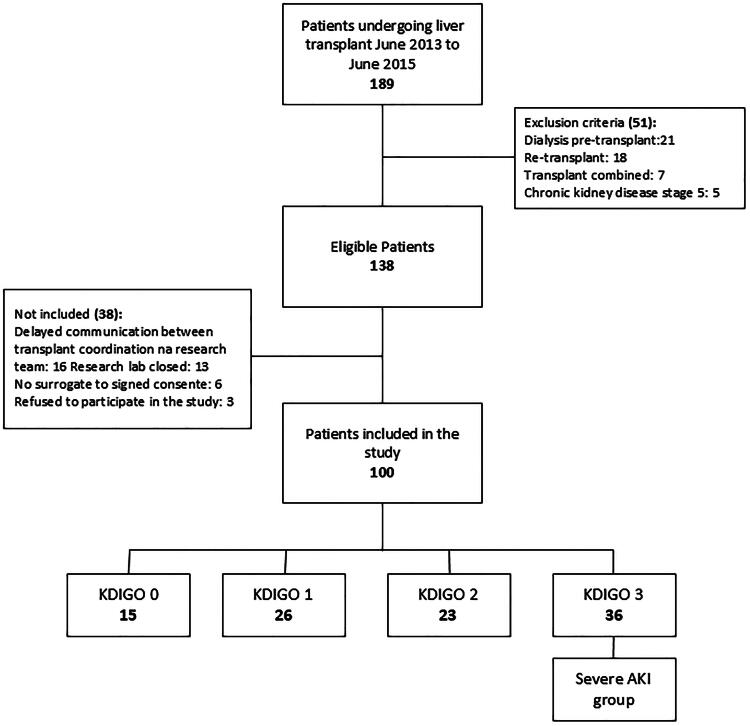
Flowchart of enrolled patients.

A total of 189 LTs were performed during the study period. 138 patients were eligible, with 100 enrolled in the study (Figure 1). The demographics and clinical characteristics of the patients are shown in Table 2. The median age was 58 ± 12 years, 64 patients (64%) were male, and the main comorbidities were hypertension and diabetes mellitus. The main causes of liver failure were hepatitis C (46%), followed by alcoholic cirrhosis (13%) and cryptogenic cirrhosis (12%). The predominant donor type was deceased after brain death (99 cases), with one case of living donation. Of the 100 patients included, 36 (36%) developed severe AKI according to the KDIGO 3 criteria and a total of 34 patients needed KRT in the first week after LT.

**Table 2.** Clinical characteristics and outcomes in patients with and without moderate to severe AKI progression.

Patient characteristics and outcomes	Total	Non-severe AKI	severe AKI	P
N	100 (100%)	64 (64%)	36 (36%)	<0.0001
Baseline Characteristics				
Age	58 (SD 12)	56.80 (SD 12.81)	54 (SD 11.13)	0.12
Gender (M)	64 (64%)	42 (61.8%)	22 (68.8%)	0.49
BMI	26 (SD 4)	25.67 (SD 3.14)	26.05 (SD 5.18)	0.48
Non caucasian	14 (14%)	10 (14.7%)	04 (12.5%)	0.81
MELD functional	15 (IQR 11–19)	13 (IQR 10–17)	16 (IQR 13–22)	0.01
MELD LT	29 (IQR 29–29)	29 (IQR 29–29)	29 (IQR 29–29)	0.96
Liver Disease				
hepatitis C	46 (46%)	32(47.1%)	14(43.8%)	0.44
Alcoholic cirrhosis	13 (13%)	09 (13.2%)	04 (12.5%)	0.68
Cryptogenic cirrhosis	12 (12%)	07 (10.3%)	05 (15.6%)	0.96
acute hepatitis	06 (06%)	05 (7.4%)	01 (3.1%)	0.64
hepatitis B	04 (04%)	03 (4.4%)	01 (3.1%)	0.70
Other	19 (19%)	12 (17.6%)	07 (21.9%)	0.91
Comorbidities				
Hypertension	33(33%)	26 (38.8%)	07 (23.3%)	0.13
Diabetes mellitus	28 (28%)	19 (28.4%)	09 (30%)	0.86
Kidney function				
baseline sCr	0.77 (IQR 0.63–0.99)	0.77 (IQR 0.62–0.98)	0.77 (IQR 0.65–1.00)	0.87
reference sCr	0.78 (IQR 0.62–1.02)	0.77 (IQR 0.64–1.02)	0.80 (IQR 0.61–1.00)	0.96
Estimated GFR(CKD-EPI) by Scr ref.	78.65 (IQR 52–99)	93.50 (IQR 64–105)	69.40 (IQR 45–98)	0.013
Estimated GFR(CKD-EPI) by Scr base	99.65 (IQR 74–110)	99.25 (IQR 75 − 108)	108.52 (69–117)	0.67
Urine output first day after LT	475 (SD45)	608 (SD79)	383 (SD49)	0.01
Fluid balance first day after LT	+1535 (IQR + 500–+2315)	+1010 (IQR + 367–+1782)	+1655 (IQR + 890–+2447)	<0.01
Severity score indices				
SAPS	64 (IQR 60–72)	62 (IQR 53–73.20)	67.50 (IQR 61–76)	<0.01
SOFA	13 (IQR 11–15)	12 (IQR 9–13)	14 (IQR 12–16)	0.001
Process of care				
Anesthesia duration (hh:mm)	09:56 (SD 01:59)	09:08 (SD 01:33)	10:32 (SD 02:04)	<0.0001
HEPATECTOMY duration (HH:MM)	03:11 (00:54)	02:54 (00:46)	03:27 (00:57)	<0.01
warm ischemia duration (HH:MM)	00:42 (1:11)	00:41 (1:21)	00:43 (1:52)	<0.0001
cold ischemia duration (HH:MM)	06:04 (01:55)	05:49 (02:06)	06:14 (01:46)	0.73
Red blood cells (unit)	2.39 (2.8)	1.45 (2.57)	3 (2.9)	<0.01
calcineurin inhibitors pre-op	39 (39%)	32 (49.2%)	07 (30.4%)	0.11
calcineurin inhibitors 48 h post-op	100 (100%)	68 (100%)	32 (100%)	0.75
Outcomes				
Time with vasoactive drugs (days)	2 (SD1.78)	1(SD 1.18)	2 (SD 2)	0.01
Days of Mechanical ventilation	2 (SD 1.82)	1 (SD 0.57)	3 (SD 2)	<0.0001
Length of ICU stay(days)	9.81 (SD 13)	5.59 (SD 6.3)	12.75 (SD 2)	<0.01
Length of hospital stay(days)	29 (SD 28)	19.17 (SD 14.6)	36 (SD 4.2)	<0.0001
Need for Re-transplantation	11 (11%)	06 (8.8%)	5 (15.6%)	0.31
Need for KRT	34 (34%)	17 (25%)	17 (53.1%)	0.006
60-day Mortality	21 (21%)	07 (10.3%)	14 (43.8%)	<0.0001

Data are expressed as n (%), mean SD (±), median and percentile (25–75) according to their distribution. Severe AKI represents patients with KDIGO 3.

BMI: body mass index; MELD: model for end-stage liver disease; LT: liver transplantation; ICU: intensive care unit; SAPS: Simplified Acute Physiology Score; SOFA: Sequential Organ Failure Assessment; KRT: kidney replacement therapy; GFR: glomerular filtration rate; CKD- EPI: Chronic Kidney Disease Epidemiology Collaboration.

### Predictors of severe AKI, and need for KRT in pre-LT assessment

There was no statistical difference in the pre-op levels of BMs based on severe AKI occurrence in post-op LT (Table 3). U-NGAL, L-FABP, and U-MCP-1 measured before surgery were good predictors of KRT need (Table 4). U-NGAL was significantly higher in the KRT group with a median of 159 (P25–75: 49–435) ng/ml versus 28 (P25–75: 17–127) ng/ml in the non-KRT group (*p* < 0.01), AUC 0.71 (CI 0.59–0.84). Lower preoperative L-FABP values were associated with the need of KRT (*p* < 0.01). Lastly, U-MCP-1 showed markedly higher levels in KRT patients; median 752 (P25–75: 364–413) pg/ml versus 354 (P25–75: 149–768) pg/ml in the non-KRT group (*p* = 0.02), AUC 0.68 (CI 0.54–0.81).

**Table 3.** Levels of pre- and post-operative biomarkers and area under curve according to AKI group in the first week after liver transplantation.

Pre-op.	N	Non severe AKI	Severe AKI (KDIGO 3)	p	AUC	Pos-op.	N	Non severe AKI	Severe AKI (KDIGO 3)	p	AUC
P-CYS (mg/l)	53	0.64 (0.38–1.36)	0.98 (0.55–1.60)	0.13	0.62 (0.47–0.78)	P-CYS (mg/l)	20	0.45 (0.36–0.56)	1.10 (0.46–2.50)	0.08	0.75 (0.50–0.99)
P-PENK (pmol/l)	57	83 (49– 130)	82 (61–122)	0.46	0.56 (0.41–0.71)	P-PENK (pmol/l)	57	94 (62– 207)	159 (122–297)	0.01	0.70 (0.56–0.84)
U-KIM-1 (pg/ml)	71	1.58 (0.60–6.11)	2.05 (0.60–3.87)	0.38	0.44 (0.30–0.57)	U-KIM-1 (pg/ml)	96	5.18 (2.07–13.16)	5.39 (3.98–14.51)	0.065	0.54 (0.42–0.66)
U-NGAL (ng/ml)	98	43 (19– 247)	66 (18–356)	0.99	0.50 (0.35–0.65)	U-NGAL (ng/ml)	98	210 (75– 1580)	1031 (915–3772)	0.05	0.62 (0.50–0.74)
P-NGAL (ng/ml)	100	210 (107–387)	167 (113–217)	0.18	0.42 (0.30–0.53)	P-NGAL (ng/ml)	100	329.5 (222.75–695.75)	406.5 (301.50–705.25)	0.98	0.56 (0.44–0.68)
U-IL-18 (pg/ml)	36	129 (77–295)	147 (100–303)	0.65	0.65 (0.35–0.74)	U-IL-18 (pg/ml)	36	237 (105–547)	760 (271–1465)	0.04	0.74 (0.53–0.94)
U-CYS ‎/cr (mg/g)	66	0.11 (0.14–41)	0.07 (0.05–0.21)	0.43	0.44 (0.30–0.58)	U-CYS/cr (mg/g)	34	0.13 (0.14–41)	0.18 (0.23–41)	0.61	0.46 (0.32–0.61)
U-GST-pi (pg/ml)	71	23.35 (15.35–53.88)	22.65 (11.30–30.61)	0.269	0.42 (0.28–0.56)	U-GST-pi (pg/ml)	71	23.35 (15.35–53.88)	22.65 (11.30–30.61)	0.264	0.42 (0.28–0.56)
L-FABP (pg/ml)	71	49.43 (11.81–215.12)	36.37 (6.37–155.82)	0.614	0.46 (0.32–0.61)	L-FABP (pg/ml)	71	49.43 (11.81–215.12)	36.37 (6.37–155.82)	0.61	0.46 (0.32–0.61)
U- a-1M ‎/cr (mg/g)	43	8.54 (2.04–56)	11.26 (3.34–77.56)	0.36	0.60 (0.40–0.80)	U- a-1M/cr (mg/g)	43	39 (3.06–105)	49 (12.22–94.28)	0.69	0.54 (0.34–0.74)
U-ALB ‎/cr (mg/g)	42	10.29 (3.67–22.51)	5.61 (0.98–11.96)	0.18	0.37 (0.17–0.56)	U-ALB/cr (mg/g)	42	22 (9.41–32.20)	33.71 (20.62– 92.65)	0.035	0.704 (0.51–0.88)
FePCS (%)	38	10.99 (6.71–39.17)	22.55 (1.05–43.27)	0.38	0.63 (0.43–0.84)	FePCS (%)	37	6.70 (0.21–13.36)	10.36 (0.64–25. 16)	0.219	0.63 (0.43–0.84)
FeHA (%)	38	433.39 (113–975)	851 (337–1102)	0.19	0.626 (0.44–0.81)	FeHA (%)	37	35.95 (6.97–619.4)	108.94 (24.97–216.96)	0.78	0.47 (0.28–0.66)
FeNa (%)	74	0.54 (0.24–0.83)	0.30 (0.06–0.62)	0.25	0.35 (0.21–0.49)	FeNa (%)	74	0.28 (0.17–0.53)	0.25 (0.10–0.44)	0.57	0.54 (0.40–0.69)
FeU (%)	71	32.42 (18.90–47.46)	29.04 (15.49–38.33)	0.55	0.45 (0.27–0.63)	FeU (%)	71	11.44 (6.76–35.44)	6.47 (1.87–16 .84)	0.01	0.68 (0.55–0.82)
U-OSM (mOsm/l)	46	470 (418– 689)	469 (343–469)	0.26	0.80 (0.67–0.93)	U-OSM (mOsm/l)	46	525 (400– 725)	355 (337–401)	0.001	0.80 (0.67–0.93)
U-MCP-1 (pg/ml)	63	449.5 (227–1256)	728 (219–2417)	0.281	0.68 (0.58–0.84)	U-MCP-1 (pg/ml)	63	727 (320–1623)	1654 (488–5388)	0.021	0.68 (0.58–0.84)
U-TIMP-1 (pg/ml)	71	6.10 (2.23–15.38)	6.81 (1.75–12.38)	0.23	0.48 (0.33–0.62)	U-TIMP-1 (pg/ml)	71	16.37 (5 .03–334)	21.90 (11.69–425.81)	0.23	0.58 (0.46–0.70)
U-UMOD (pg/ml)	35	260 (107.5–445)	302.5 (90.25–448)	0.99	0.50 (0.30–0.69)	U-UMOD (pg/ml)	35	64 (45.50–117)	77.50 (44.25–144)	0.10	0.53 (0.33–0.73)

P: plasma; U: urinary; CYS: cystatin; PENK: pro-enkephalin; IL-18: interleukine-18; KIM-1: kidney-injury-molecule; NGAL: neutrophil gelatinase-associated lipocalin; a-1M: alpha 1 microglobulin; L-FABP: liver-type fatty acid-binding protein; GST-pi: glutathione S-transferases; ALB: albumin; (Fe): fraction excretion; Na: sodium; U: urea; PCS: p-creasol sulfate; HA: hippurate; OSM: osmolality; MCP-1: monocyte chemotactic protein-1;– TIMP-1: tissue inhibitor of metalloproteinase 1; UMOD: uromodulin.

**Table 4.** Levels of pre- and post-operative biomarkers and area under curve according to KRT need in the first week after liver transplantation.

Pre-op.	N	No KRT	Need of KRT	P	AUC	Pos-op.	N	No KRT	Need of KRT	P	AUC
P-CYS (mg/l)	53	0.80 (0.61–1.30)	0.73 (0.36–1.56)	0.71	0.470 (0.30–0.64)	P-CYS (mg/l)	20	0.45 (0.33–0.91)	0.55 (0.43–1.78)	0.678	0.56 (0.29–0.83)
P-PENK (pmol/l)	57	67.27 (51.75–106.40)	100 (65.10–162.03)	0.10	0.631 (0.47–0.78)	P-PENK (pmol/l)	57	94 (62–159)	194 (143–311)	0.001	0.76 (0.63–0.89)
U-KIM-1 (pg/ml)	71	1.58 (0.60–5.82)	2.06 (0.58–3.77)	0.76	0.479 (0.34–0.62)	U-KIM-1 (pg/ml)	96	5.27 (1.78–15.27)	5.24 (3.49–12.76)	0.565	0.54 (0.42–0.66)
U-NGAL (ng/ml)	98	28 (17–127)	159 (49–435.25)	0.003	0.71 (0.59–0.84)	U-NGAL (ng/ml)	98	135 (51–507)	1559 (473–3917)	<0.0001	0.80 (0.72–0.89)
P-NGAL (ng/ml)	100	172 (102–307)	209 (115.75–301.25)	0.39	0.55 (0.43–0.68)	P-NGAL (ng/ml)	100	287.5 (191–438)	669 (368–1662)	<0.0001	0.80 (0.71–0.88)
U-IL-18 (pg/ml)	36	123 (69.75–279.5)	192.5 (92–333.25)	0.31	0.60 (0.42–0.79)	U-IL-18 (pg/ml)	36	214.5 (119–412)	1116.5 (298–1682)	0.002	0.80 (0.64–0.95)
U-CYS ‎/cr (mg/g)	66	0.85 (0.05–41)	0.69 (0.21–10.43)	0.75	0.476 (0.33–0.62)	U-CYS/cr (mg/g)	34	0.18 (0.01–0.33)	0.10 (0.002–0.44)	0.736	0.54 (0.34–0.74)
U-GST-pi (pg/ml)	71	13.1 (23–50)	22.19 (12–42)	0.61	0.63 (0.50–0.75)	U-GST-pi (pg/ml)	71	43 (24–247)	823 (29–1446)	0.041	0.63 (0.50–0.75)
L-FABP (pg/ml)	71	67.76 (19–234)	8.94 (4.68–79.09)	0.004	0.29 (0.16–0.43)	L-FABP (pg/ml)	71	212 (51–932)	924 (157–3193)	0.012	0.66 (0.54–0.77)
U- a-1M ‎/cr (mg/g)	43	9.20 (2.98–49.36)	26 (2.95–144)	0.37	0.594 (0.37–0.81)	U- a-1M/cr (mg/g)	43	31 (6.38–94)	71.66 (22.57– 107)	1.22	0.65 (0.47–0.83)
U-ALB ‎/cr (mg/g)	42	9.53 (3.70–24.35)	5.87 (0.71–14.28)	0.21	0.37 (0.18–0.57)	U-ALB/cr (mg/g)	42	22.61 (9.22–30.15)	44.58 (19.50–111)	0.122	0.65 (0.47–0.83)
FePCS (%)	38	10.99 (5.03–32.97)	26 (2.29–84.23)	0.69	0.54 (0.33–0.75)	FePCS (%)	37	7.76 (0.11–14.64)	6.93 (2.58–21.85)	0.34	0.60 (0.40–0.78)
FeHA (%)	38	520 (128–922)	816 (193–1.520)	0.47	0.572 (0.38–0.77)	FeHA (%)	37	32.33 (6.71–532.67)	192.5 (98.28–1219)	0.06	0.64 (0.52–0.87)
FeNa (%)	74	0.55 (0.21–0.80)	0.35 (0.17–0.62)	0.26	0.416 (0.28–0.55)	FeNa (%)	74	0.23 (0.11–0.59)	0.31 (0.22–0.39)	0.587	0.46 (0.33–0.60)
FeU (%)	71	33.92 (23.44–46.06)	26.21 (11.37–46.87)	0.25	0.403 (0.22–0.58)	FeU (%)	71	13.38 (6.59–38.91)	7.26 (2.84–12.15)	0.018	0.67 (0.54–0.79)
U-OSM (mOsm/l)	46	471 (394–701)	467 (386–637)	0.78	0.473 (0.29–0.66)	U-OSM (mOsm/l)	46	534 (420–725)	338 (331–354)	<0.0001	0.98 (0.95–1.00)
U-MCP-1 (pg/ml)	63	354 (149–768)	752 (364–413.5)	0.02	0.675 (0.54–0.81)	U-MCP-1 (pg/ml)	63	488 (232–1447)	3976.50 (727–5371)	<0.0001	0.80 (0.69–0.92)
U-TIMP-1 (pg/ml)	71	5.66 (1.90–12.88)	7.10 (1.82–18.93)	0.94	0.506 (0.36–0.65)	U-TIMP-1 (pg/ml)	71	16.29 (5.03–425.81)	25.99 (12.22–425.81)	0.139	0.59 (0.48–0.71)
U-UMOD (pg/ml)	35	260 (108–445)	303 (90.2–448)	0.987	0.498 (0.30–0.70)	U-UMOD (pg/ml)	35	78 (42.5–143)	64 (42–122)	0.755	0.53 (0.34–0.73)

KRT: kidney replacement therapy; P: plasma; U: urinary; CYS: cystatin; PENK: pro-enkephalin; IL-18: interleukine-18; KIM-1: kidney-injury-molecule; NGAL: neutrophil gelatinase-associated lipocalin; a-1M: alpha 1 microglobulin; L-FABP: liver-type fatty acid-binding protein; GST-pi: glutathione S-transferases; ALB: albumin; (Fe): fraction excretion; Na: sodium; U: urea; PCS: p-creasol sulfate; HA: hippurate; OSM: osmolality; MCP-1: monocyte chemotactic protein-1; TIMP-1: tissue inhibitor of metalloproteinase 1; UMOD: uromodulin.

### Predictors of severe AKI, and need for KRT in post-LT assessment

When assessed within 48 h post-surgery, several BMs were good predictors of the risk for progression to KDIGO 3 in the first week after LT: P- PENK, U-NGAL, U- IL-18, U-ALB, FeU, U-OSM, and U-MCP-1 (Table 3). P-PENK, U-NGAL, P-NGAL, U-IL-18, U- GST-pi, U-L-FABP, FeU, U-OSM, and U-MCP-1 were good predictors of KRT need (Table 4).

P-PENK was significantly higher in patients that developed severe AKI, with a median 94 (P25–75: 62–207) pg/ml in non-severe AKI versus 159 (P25–75: 122–297) pg/ml in severe AKI group, with an AUC 0.70 (CI 0.56 − 0.84), *p* = 0.01 (Table 3). P- PENK had a median 94 (P25–75: 62–159) pg/ml in non-KRT versus 194 (P25–75: 143–311) pg/ml in KRT patients, with an AUC of 0.76 (CI 0.63–0.89), *p* = 0.001 (Table 4).

U-NGAL was significantly higher in patients that developed severe AKI, with a median of 210 (P25–75: 75–1580) ng/ml in non-severe AKI versus 1031 (P25–75: 915–3772) ng/ml in severe AKI group; AUC 0.62 (CI 0.50 − 0.74), *p* = 0.05 and remained markedly elevated in KRT patients’ group, with an AUC 0.80 (CI 0.72–0.89), *p* < 0.0001. U-IL-18 showed a good performance for predicting both outcomes, severe AKI and need of KRT, with an AUC 0.74 (CI 0.53–0.95), *p* = 0.04 and 0.80 (CI 0.64–0.95), *p* = 0.002, respectively (Table 4).

Tubular stress was assessed using the enzymes U-L-FABP and U-GST-pi; both effectively predicted the need for KRT 48 h after LT. U-L-FABP had higher expression in the KRT group with a median of 924 (P25–75: 157–3193) versus 212 (P25–75: 51–932) pg/ml in the non-KRT group, AUC 0.66 (CI 0.54–0.77), *p* = 0.012. GST-pi median was 43 (P25–75: 24–247) pg/ml in non-KRT vs 823 (P25–75: 29–1446) pg/ml for the KRT group, AUC 0.63 (CI 0.50–0.75), *p* = 0.004.

Reabsorption BM (U-ALB) showed higher expression in severe AKI group with a median of 22 (P25–75: 9.41–32.20) vs 33.71 (P 25–75: 20.62–92.65) mg/g in non-severe AKI, with an AUC 0.70 (CI 0.51–0.88), *p* = 0.035. In the same post-op 48 h period, FeU had lower levels in patients that developed severe AKI and needed KRT, with a median of 11.44 (P25–75: 6.76–35.44) % in non-severe AKI group and 6.47 (P25–75: 1.87–16.84) % in severe AKI, *p* = 0.01 and an AUC 0.68 (CI 0.55–0.82) and maintained its performance in determining KRT need with an AUC 0.67 (CI 0.54–0.79) *p* = 0.018 (Tables 3 and 4).

Concentration was analyzed by U-OSM, and it showed a good performance for predicting severe AKI: median of 355 (P25–75: 337–401) mOsm/l in severe AKI vs 525 (P25–75: 400–725) mOsm/l in non-severe AKI– AUC 0.80 (CI 0.67–0.93), *p* = 0.001. U-OSM was also remarkably useful in predicting the need for KRT with a median of 338 (P25–75: 331–354) mOsm/l vs 534 (P25–75: 420–725) mOsm/l in the non-KRT group – *p* < 0.0001, AUC 0.98 (CI 0.95–1.00) (Tables 3 and 4).

U-MCP-1 showed significantly increased levels in patients who developed severe AKI compared to the non-severe AKI group. An AUC of 0.80 (CI 0.69–0.92) was obtained for the KRT need prediction. U-UMOD, a marker of kidney function reserve, had significantly lower levels in severe AKI and KRT patients but was not statistically significant.

### Assessment of all BMs in CART algorithm

**Figure 2.**
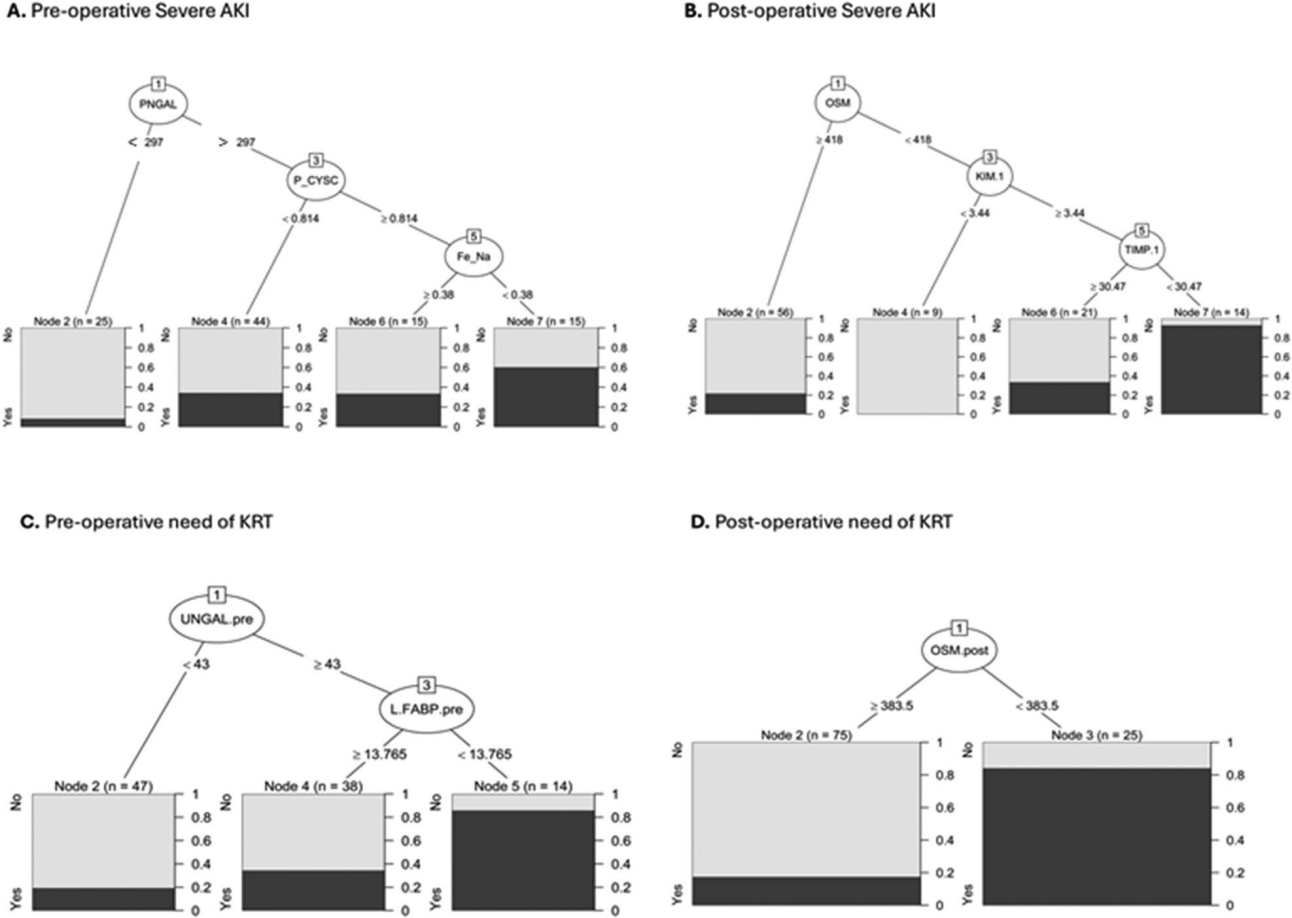
The best variables selected by the classification and regression trees (CART) model according to the outcomes. The squares (nodes) are presented in two different colors: in light gray the patients with positive outcome (No) and in black gray the patients with negative outcome (Yes). The graduation of each end classification is available in the left of the node, and the number of patients included for each node is available above the squares. The cutoff values are shown in the figure for each node. KRT: kidney replacement therapy; P: plasma; U: urinary; CYS: cystatin C; KIM-1: kidney-injury-molecule 1; NGAL: neutrophil gelatinase associated lipocalin; L-FABP: liver-type fatty acid-binding protein; Fe_Na: fraction excretion of sodium; OSM: osmolality; TIMP-1: tissue inhibitor of metalloproteinase.

The performance of the nineteen BMs was analyzed into the CART algorithm to evaluate the best predictors of severe AKI, and KRT need (Figure 2).

### Severe AKI prediction pre- and post-LT

The first split modeling by CART overall BM in pre-LT for predicting severe AKI named (node) was P-NGAL with a cutoff value of 297 ng/ml, following the child node P- CYS with a cutoff value of 0.814 and the last node FeNa with a cutoff of 0.38%. Together they achieved an accuracy of 71.72%, cross-validation error (CV 32.3%), and Cohen’s kappa of 0.24 with a fair agreement, sensitivity of 29%, specificity of 91%, and positive predictive value (PPV) of 60%, and negative predictive value (NPV) of 74%. In post-LT for predicting severe AKI, U-OSM was the best overall variable, with a cutoff value of 418 mOsm/l (Figure 2); the child nodes were U-KIM-1 following U-TIMP-1 with an accuracy together above 80%, - (CV 25%) and Cohen’s kappa 0.46 with a moderate agreement, sensitivity 41%, specificity 98.5% and PPV 93% and NPV 78%.

### KRT need prediction pre- and post-LT

The first split modeling by CART overall BM in pre-LT for predicting KRT need was U-NGAL, following the child node U-L-FABP and together they showed a sensitivity of 35.29% and a specificity of 96.92%, with an accuracy of 75.76%, - (CV 34.34%) and Cohen’s kappa 0.46 with a moderate agreement, sensitivity 35.3%, specificity 96.9% and PPV 85.7% and NPV 74%. In the post-LT, the U-OSM showed the best discrimination for KRT need; a value higher than 383.5 mOsm/l had an accuracy of 83% - (CV 26%), and Cohen’s kappa 0.60 with good agreement, sensitivity 62%, specificity of 94% and PPV 84% and NPV 83%.

## Discussion

This study evaluated nineteen BMs representing different aspects of kidney function, such as secretion, concentration, kidney reserve, and fibrosis, which were rarely previously studied in this acute setting. A comprehensive assessment of kidney function, beyond injury and filtration, allows a more robust and detailed AKI monitoring Moreover, both concentration BM (U-OSM) and injury BM (U-NGAL) were demonstrated in this study to be relevant predictors of both severe AKI and need of KRT when measured early in the postoperative period.

Kidney reserve BMs, in turn, can more accurately indicate the impact of future injuries due to interventions, such as LT, and can signal kidney disease progression through fibrosis BMs.

Based on prior studies [14–21], we have selected a combination of BMs to compose a comprehensive kidney function evaluation that simultaneously quantifies tubular function and injury components. P-PENK is a newer BM of glomerular filtration impairment, and unlike P-CYS, it is not influenced by inflammatory processes [24,25]. Previous studies have evaluated P-PENK ability to predict AKI in different settings, but few have focused on cirrhosis or LT [21]. In sepsis, P-PENK has demonstrated good prediction ability for severe AKI and sepsis development [26–29]. In our study, P-PENK level measured 48 h after LT was a good predictor of severe AKI development and KRT need.

Injury BMs have been widely studied in the literature in various clinical scenarios [30–32], and NGAL is the most frequently analyzed. In our study, U-NGAL showed better results than P-NGAL for predicting severe AKI and KRT needs. The best ability can be explained because U-NGAL is released first in the urine and similar results were found in previous studies [20,33]. In our cohort, some patients had higher levels of U-NGAL before surgery in severe AKI. This finding (pre-surgery) was also found in children undergoing cardiopulmonary bypass [34] and in a meta-analysis [35]. In the CART model, U-NGAL, in addition to U-OSM and U-TIMP-1, were the predictors of choice for determining KRT need in pre-LT. This combination achieved an accuracy of 80%. Notwithstanding, P-NGAL was useful in CART in pre-LT to determine severe AKI, with an accuracy of 71.72%, together with P-CYS and FeNa (Figure 2).

Additionally, the inflammatory BM U-IL-18 showed good discrimination between severe AKI and non-severe AKI when measured early in the postoperative period (Table 3). This aligns with findings from other studies, such as those by Sirota et al. [17], who also observed higher expression of IL-18 and NGAL in AKI patients post-LT. Contrary to many studies, our research did not find U-KIM-1 or U-CYS to be predictive of severe AKI and KRT in LT. Tsuchimoto et al. [36] also failed to predict AKI using U-CYS in the serial analysis of thirty-one LT recipients, including pre- and post-operative assessment.

The study also highlighted that tubular secretion represents a vital homeostatic function for rapidly clearing endogenous solutes and drug elimination from the circulation [37]. Studies reveal that the fractional excretion of HA, cinnamoylglycine, indoxyl sulfate, and PCS have been shown to have only a moderate correlation with measured (m) glomerular filtration rate (GFR), even though it was associated with fractional excretion of electrolytes [37,38]. In patients with CKD, the lower clearance of HA and PCS was associated with mortality in 3 years, independent of estimated GFR [39]. In our study, FeHA and FePCS were not predictors of severe AKI or KRT need. The evaluation of these BMs in the acute setting is very limited, leading to a lack of knowledge about adequate therapeutic doses, particularly antibiotic therapy, in which overestimating kidney function can lead to nephrotoxicity.

**Figure 3.**
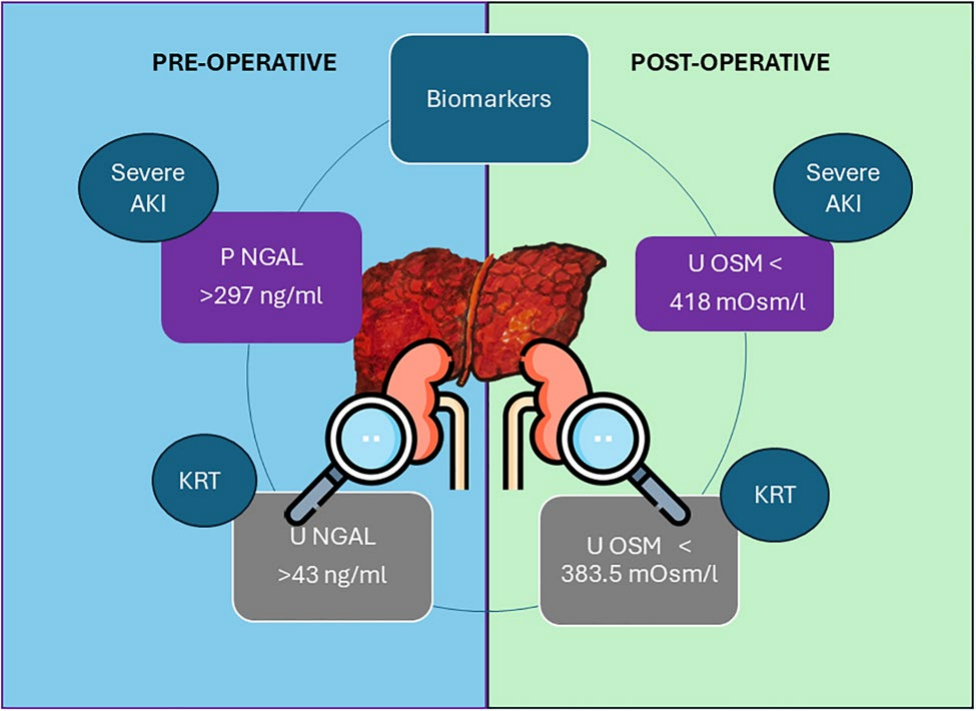
Highlighted biomarkers assessed pre and postoperatively for predicting severe AKI diagnosis and need of KRT. Based on the findings from our study, we recommend analyzing pre-operative plasma NGAL levels to predict severe AKI, with values above 297 ng/ml indicating risk. For pre-operative urinary NGAL, levels exceeding 43 ng/ml suggest the post-operative necessity of KRT. In the post-operative period, we propose the following thresholds: a U-OSM value lower than 418 mOsm/l for severe AKI and lower than 383.5 mOsm/l for KRT need.

Currently, most BMs investigated in this study are unavailable for routine clinical practice use. However, the U-OSM, U-ALB, and FeU are available in clinical practice, and exploring its applicability may add an important tool to the overall evaluation of kidney function. Our study demonstrated an excellent performance of U-OSM in post-op to predict severe AKI. U-OSM was the first variable of choice by CART and, in addition to U-KIM-1 and U-TIMP-1, showed an accuracy of 80%. U-OSM was also the best variable by CART for predicting KRT need, with an accuracy of 83%. Corroborating with our findings, low osmolality was previously demonstrated as an independent risk factor for CKD progression [40,41]. Our study suggests, however, that U-OSM may be a relevant single parameter to be monitored post-operatively in LT (Figure 3), especially with real-time assessment models in which the early loss of urinary concentration capability may be an alert sign of subclinical AKI development. Preoperatively, NGAL measured in plasma and urine may serve as a single BM to be assessed with relevant prognostic information.

Urinary biochemistry evaluation using FeNa and FeU, classic BMs used to differentiate intrinsic AKI from prerenal AKI, is also available [42]. Recently, Patidar et al. [43] retrospectively evaluated FeU in 50 patients with cirrhosis and found that FeU showed good results in distinguishing structural from functional AKI. FeU had good performance for predicting severe AKI with an AUC of 0.68 (CI 0.54–0.82), and KRT was needed with an AUC of 0.67 (CI 0.54–0.74).

Our study has some limitations. Firstly, we could not measure all biomarkers in the entire cohort due to budget constraints. Secondly, our patients had an elevated median MELD score of 29. Thus, similarly to previous studies [33,44], we had a high proportion of patients with AKI (85%) and severe AKI (36%). As a result, the ability of these biomarkers to predict non-severe AKI was not assessed. Additionally, the lack of data on creatinine 60 days after LT and CNI levels and the absence of long-term data about kidney function limits our ability to evaluate the impact of biomarkers on post-LT CKD. To validate our findings, it is essential to conduct assessments in larger cohorts that encompass less severely ill patients.

This study also possesses notable strengths. The prospective analysis ensured consistent data collection, and the diagnosis of AKI was conducted over 7 days, a longer duration than most studies that typically limit evaluation to 48 h [14–19]. This extended analysis period provides a more realistic depiction of post-operative AKI incidence. Addressing concerns about late AKI diagnosis based on sCr, including three key biomarkers (P-PENK, U-OSM, and U-TIMP-1) in CART analysis, enables the early prediction of severe AKI. Prediction of the need for KRT was feasible with two biomarkers (U-NGAL and U-L-FABP), even before surgery. Lastly, to our knowledge, no prior studies have comprehensively analyzed all aspects of kidney health, including filtration, secretion, tubular reserve, and fibrosis, in both the pre- and post-operative stages of LT.

## Conclusion

This study demonstrated the potential to enhance the prediction of severe AKI and the need of KRT in individuals undergoing LT incorporating biomarkers related to filtration, tubular injury, and concentration. A more comprehensive evaluation of kidney function through biomarkers could open avenues for gaining new insights into the causes of AKI, fostering innovative diagnostic methods, and supporting novel therapeutic approaches, essential for advancing research in critically ill patients. U-OSM has emerged as a viable alternative in this cohort to determine the severity outcomes of AKI and the need of KRT after LT and therefore could be further explored in clinical practice and in future studies.

## Data Availability

The datasets used in this study are not publicly available because of confidentiality. However, it can be made available by the corresponding author Camila Lima upon reasonable request.
